# Testicular aging, male fertility and beyond

**DOI:** 10.3389/fendo.2022.1012119

**Published:** 2022-10-13

**Authors:** Shijue Dong, Chen Chen, Jiali Zhang, Yuan Gao, Xuhui Zeng, Xiaoning Zhang

**Affiliations:** ^1^ Institute of Reproductive Medicine, Medical School, Nantong University, Nantong, China; ^2^ Laboratory Animal Center, Nantong University, Nantong, China

**Keywords:** male aging, testicular premature aging, spermatogenesis, sperm function, aging-dependent infertility

## Abstract

Normal spermatogenesis and sperm function are crucial for male fertility. The effects of healthy testicular aging and testicular premature aging on spermatogenesis, sperm function, and the spermatogenesis microenvironment cannot be ignored. Compared with younger men, the testis of older men tends to have disturbed spermatogenic processes, sperm abnormalities, sperm dysfunction, and impaired Sertoli and Leydig cells, which ultimately results in male infertility. Various exogenous and endogenous factors also contribute to pathological testicular premature aging, such as adverse environmental stressors and gene mutations. Mechanistically, Y-chromosomal microdeletions, increase in telomere length and oxidative stress, accumulation of DNA damage with decreased repair ability, alterations in epigenetic modifications, miRNA and lncRNA expression abnormalities, have been associated with impaired male fertility due to aging. In recent years, the key molecules and signaling pathways that regulate testicular aging and premature aging have been identified, thereby providing new strategies for diagnosis and treatment. This review provides a comprehensive overview of the underlying mechanisms of aging on spermatogenesis. Furthermore, potential rescue measures for reproductive aging have been discussed. Finally, the inadequacy of testicular aging research and future directions for research have been envisaged to aid in the diagnosis and treatment of testicular aging and premature aging.

## Introduction

**Figure 1:**
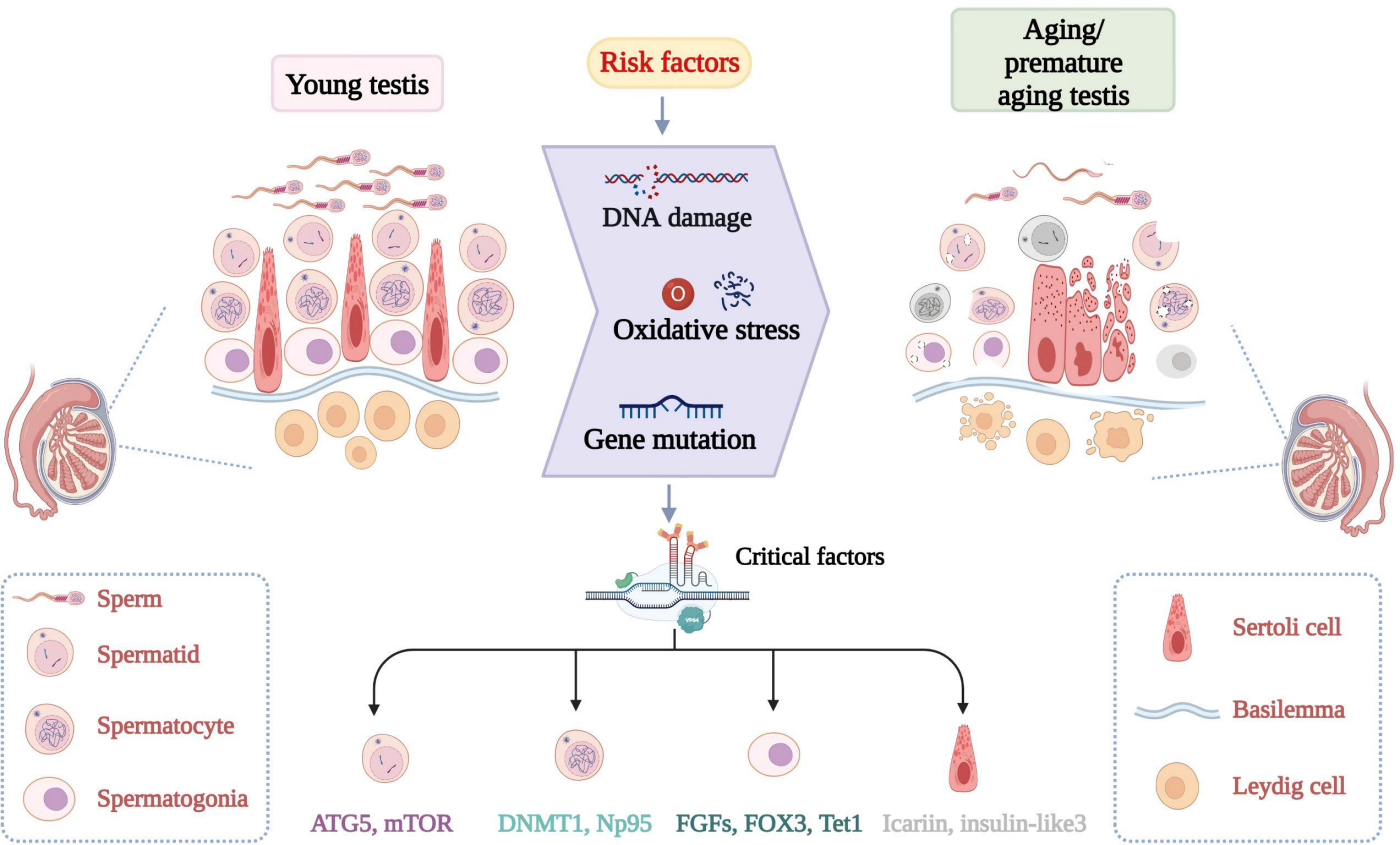
Discrepancy of normal spermatogenesis and senescent spermatogenesis. In young testes, spermatogenesis continues through a series of specific steps. In aging testes, spermatogenesis is impaired with age. Increased risk factors and critical molecular aberrant cause adverse changes in the morphology and number of germ cells. Changes in key molecules such as FOX3 can lead to testicular DNA damage, increased oxidative stress and so on.

The arrival of the information era and the rise in socioeconomic levels have exacerbated the life pressures and childbearing costs, and it has become increasingly common for both men and women to voluntarily delay having children (1). Not only did the proportion of women over the age of 50 who had no children rise from 13.6% in 1989 to 19.6% in 2016 in Finland (2) but also the men age had their first child rose from 27.4 years in 1972 to 30.9 years in 2015 in the United States (3). However, delaying childbirth has led to infertility issues among many elderly couples or the inability to have a second or third child. The frequent occurrence of these phenomena has raised concerns related to the impact of age and premature senescence on fertility and reproductive risks. It is generally believed that the negative effects related to reproduction caused by increased parents’ age are mainly attributable to women, and few studies evaluate the consequences of the increase in paternal age. However, there is growing evidence that the increase in male age has a significantly negative impact on spermatogenesis, sperm function, fertilization, pregnancy, and offspring health (4, 5). Germ cell loss and impaired spermatogenesis due to testicular aging have been observed in elderly men as well as in other elderly mammals (6, 7). The numbers of germ cells in the seminiferous tubules have been shown to decrease with age, which results in the shrunken diameter of the seminiferous tubules (8) and the vacuolization of the seminiferous epithelium (9). Some studies have reported adverse changes in semen parameters and sperm morphology with the increase in male age, including the decrease in semen volume and sperm motility as well as increase in sperm deformity rate (10–12). The effects of paternal aging on semen parameters and reproductive health have been well studied (13, 14). Moreover, the spermatogenesis microenvironment formed by the Sertoli and Leydig cells exhibits significant abnormalities with age, such as decreased number, morphological variations, organelle aging, abnormal hormone secretion, and blood–testicular barrier defects (15). In addition to the damage caused by normal aging to male reproduction, testicular premature aging (testicular premature aging highlighted in this review refers to aging-dependent subfertility or infertility caused by multiple endogenous and exogenous factors, such as mental stress, adverse lifestyle habits, and radiation, which trigger genetic mutations in their molecular mechanisms) is associated with the phenotype of testicular senescence (testicular senescence emphasized in this review is healthy or physiological aging caused by the pure-age effect) under physiological conditions, thereby triggering a series of reproductive disorders and even infertility and health risks (**Figure 1**). This phenomenon has gradually attracted clinical attention. More importantly, studies on reproductive premature aging performed in recent years have not been systematically reviewed. It is also necessary to investigate how these adverse effects can be reduced to make pertinent predictions or alleviate fertility risks and assist healthy human reproduction and eugenics. Hence, this article systematically reviews the focal issues of physiological and pathological reproductive aging as well as the corresponding prevention and treatment strategies, thereby prospectively providing assistance and serving as a reference for relevant studies in the field.

## Aging and spermatogenesis

Spermatogenesis is a process whereby the spermatogonia are first differentiated into primary spermatocytes *via* mitosis, then form spermatids *via* meiosis, and are finally transformed into highly differentiated haploid spermatozoa. The continued production of motile spermatozoa poses several requirements for spermatogenesis. First, the organism must be able to maintain a normal reproductive cycle, and have a thriving population of spermatogonia. Second, to produce sufficient haploid gametes, ongoing meiosis and the generation of spermatids are needed. Finally, the generation of elongated spermatozoa necessitates the specific transformation of spermatid morphology and the expression of key genes (16). With the increase in male age, the above processes change irreversibly to varying degrees. Although spermatogenic cells are one of the cells with the lowest spontaneous mutation rates in all human tissues (17, 18), studies have indicated that the germ cells undergo genetic mutations and abnormal spermatogenesis with advancing male age (19, 20). Germ cell loss and impaired spermatogenesis have been observed in older men and other mammals, particularly during spermatogonia mitosis, spermatocyte meiosis, and sperm deformation. Germ cell apoptosis increases, the capacity of the cells to resist oxidative damage decreases, and abnormal metabolism appear with increasing paternal age (16). In addition, the frequency of genetic mutations is increased, and epigenetic modifications occur in the germ cells (21). Recently, key molecules and signaling pathways involved in testicular aging and premature aging have been gradually revealed with the use of multiple technologies, including transcriptomics and proteomics, and evidence from a large number of knockout (KO) animal models. Moreover, attempts have been made to apply these molecular targets to investigate strategies or drugs against reproductive aging (22, 23).

A recent report has profiled the whole transcriptome of aging mouse testes to reveal the gene transcriptional landscape underlying male reproductive aging. The study found that several testis-specific or predominant expression transcripts are strongly associated with male reproductive aging. Age-related expression of lncRNAs reflected cellular senescence to some extent. The researchers then analyzed the potential cis-regulatory targets of these lncRNAs, such as *Agtpbp1*, *Suds3*, *2410089E03Rik*, *4921509C19Rik*, and *Tex14*, whose expression levels are linked to age and reproduction (24). TEX14 has previously been shown to be an essential molecule for the formation of intercellular bridges among spermatogonia and for male mouse fertility (25). Thus, lncRNAs may affect age-dependent fertility in mice by regulating *Tex14* expression. In addition to lncRNAs, miRNA expression profiles undergo modifications with age, thereby playing important roles in male reproductive aging. A study has shown that miR-122 expression is reduced five-fold in older males. Furthermore, miR-122a, miR-371-3p, miR-19b, and miR-146a have been implicated in the regulation of aging and spermatogenesis (12). Hence, in the context of male reproductive aging, it is necessary to conduct an in-depth study on how noncoding RNAs regulate testicular aging and premature aging to comprehend the underlying mechanisms. Proteomic studies on nonhuman primates using testis tissues have suggested that many proteins are differentially expressed in the testicular peritubular cells and testis tissues of the aging group compared with the young group. The expressions of several proteins, such as CDKN2A with antiproliferative function and CNN1 with cellular contractile function, are upregulated in aging testes, which suggests their role in promoting testicular senescence. Further analysis revealed that the functions of aging testicular peritubular cells are significantly altered with regard to protein secretion, NF- κB signaling, and stretch functions. These omics findings have provided numerous candidate molecules to further probe the mechanisms of testicular aging and premature aging (26, 27).

Despite extensive investigation, the fine mechanisms by which aging leads to these alterations remain poorly defined in spermatogenesis. Recent studies have alluded that the paternal age effect leads to germline abnormalities that result from the continuous accumulation of germline mutations over time (28). The underlying molecular mechanisms include microdeletions of the Y chromosome (29), increase in telomere length (30), accumulation of DNA damage with reduced repair capacity (31), oxidative stress (32), gene mutations (33), and alterations in epigenetic modifications, such as DNA methylation (34). These findings signify that not only should the child-bearing age of women be optimal (18–35 years) but also the reproductive age of men should be appropriate to reduce the occurrence of adverse effects. Findings from animal models suggest that mutations in many genes can lead to testicular premature aging or even infertility, whereas normal expression or overexpression of key genes helps maintain normal physiological functions of the testis. For instance, a transcriptional coactivator with PDZ binding motif (TAZ) relieves P53-induced transcription of *p21* mRNA by inhibiting the DNA binding ability of P53, which, in turn, inhibits apoptosis and the premature senescence of spermatogenic cells (35). More reports on testicular premature aging are presented in **Table 1**. In addition, male fertility disorders related to testicular premature aging are caused by the stress of exogenous adverse factors. For example, exposure to environmental endocrine disruptors decabromide diphenyl ether and di-(2-ethylhexyl) phosphate (DEHP) causes age-dependent premature reproductive senescence in male mice (56, 57). A study shows that testicular aging is closely related to body mass index (BMI) in the elderly. Older men with higher BMI have more severe testicular aging changes (58). Elucidating the mechanisms leading to reproductive senescence under these physiological or pathological conditions can facilitate the development of precisely targeted therapies for pathological testicular premature aging by detecting or intervening at key nodes in the future.

**Table 1:** The roles of reproductive senescence regulatory genes.

References	Genes	Fecundity in KO mice and specific role of genes
(36)	*Gdi1*	Infertility older than 12-week-old; Smaller diameters and more vacuoles were in seminiferous tubules in 20-week-old KO mice
(37)	*Cdk4*	Progressive subfertility; a reduced number of spermatozoa were in the 4-month-old *Cdk4^-/-^* seminiferous tubules epididymis
(33)	*Bub1b*	Infertility; Sperm counts of 4-month-old were about four times lower than WT. KO sperm produced 2-cell-stage embryos at 13 times less frequently
(38)	*Taf4b*	Infertility older than3-month-old; FSH levels increase at 8–9 weeks of age. Sperm motility and acrosome formation were impaired by 12 weeks.
(39)	*Lpar1/2/3*	Fertile; At 4 weeks of age, TKO testes showed reduction in cell numbers, along with increased vacuoles in seminiferous tubules, becoming more severe by 6 months
(40)	*Nrf2*	Progressive subfertility with increasing age; Testicular, epididymal sperm counts and sperm motility decline with age
(41)	*Gata4*	Late-onset testicular atrophy and fertility loss; By 6 months of age, KO testis weight was 59% of that control, and by 8 month of age it was 41%.
(42)	*Txndc2/3*	Fertility (3-9 months); An increase in sperm DNA damage was significant at both 12 and 18 months of age.
(43)	*Fatl3*	Fertile; Fatl3 deletion causes marked testicular enlargement in aged. Structural integrity of the testis is maintained at 16 months in KO mice compared to WT testes undergoing testicular regression.
(44)	*Jmjd1c*	Infertility after 3 months of age; At 8 months of age, *Jmjd1c*-deficient mice had smaller testes and fewer sperm with age
(45)	*Prdx6*	Progressive subfertility with increasing age; Sperm maturation and motility are impaired in *Prdx6^-/-^* during aging. Sperm from Prdx6^-/-^ mice had higher cytoplasmic droplet retention and this proportion was increased during aging
(46)	*Sod1*	Progressive decline about the number of pups per litter in *Sod^-/-^* mice; KO mice had lower body weights, testis weights, sperm counts. Young *Sod^-/-^* mice had significantly reduced tubule diameters, further serious with aging.
(47)	*Erk1/2*	Moderate subfertility with age; ERK KO males displayed significant reduction in epididymal sperm counts at 6 months of age, with a modest reduction in seminiferous tubule area. By 12 and 18 months of age, KO animals had substantially lower sperm counts and testis weights
(48)	*Bnc1*	Progressive infertility. No pup was produced after 12 weeks; Testes sizes, germ cells and serum level of testosterone displayed aging-related decline.
(49)	*Cdyl*	Progressive infertility; KO male mice suffered from oligozoospermia by 5 months. Ectoplasmic specialization, the particular cell junctions that connect the spermatids and Sertoli cells, were also disrupted
(50)	*Dicer1*	Premature aging and infertility; *Dicer1* mutant mice display highly postnatal lethality and the few survivors display accelerated aging and infertility
(51)	*eCs*	Fertility dropped with age; The litter size of *eCs*-KO males and two-cell formation in IVF was remarkably reduced in 6-month-old.
(52)	*Tet1*	Impaired fertility with age; *Tet1^-/-^* males produced markedly reduced number of pups by 11-month-old. The testis of *Tet1^-/-^* males was getting smaller with age, while WT mouse testes became slightly larger with age.
(53)	*Rora*	Subfertility; Progressive testicular damage present with the advancement of age in mutant mice. The aged mice showed a mosaic pattern of seminiferous tubules. Tubules showed atrophy, clusters of vacuoles and absence of mitotic cells.
(54)	*Csnk1g2*	Subfertility in 12-month-old KO mice; The body weight, average size and weight of the testis of 12-month-old KO mice were significantly smaller and many of their seminiferous tubules were empty.
(55)	*Ggcx*	Late-onset male infertility; The 4-month-old KO testes exhibited large multinuclear spermatids and intercellular space. Sperm concentration and hyperactive sperm was decreased but abnormal moving sperm was increased.

Gdi1, GDP dissociation inhibitor alpha; Cdk4, Cyclin-dependent kinase 4; Bub1b, Mitotic checkpoint serine/threonine kinase; Taf4b, TATA-box binding protein associated factor 4b; Lpar1, Lysophosphatidic acid receptor 1; Lpar2, Lysophosphatidic acid receptor 2; Lpar3, Lysophosphatidic acid receptor 3; Nrf2, Nuclear factor, erythroid derived 2, like 2; Gata4, GATA binding protein 4; Txndc2, Thioredoxin domain containing 2; Txndc3, Thioredoxin domain containing 3; Fatl3, Follistatin-like 3; Jmjd1c, Jumonji domain containing 1C; Prdx6, Peroxiredoxin 6; Sod1, Superoxide dismutase 1; Erk1/2, Extracellular signal-regulated kinase 1 and 2; Bnc1, Basonuclin 1; Cdyl, Chromodomain protein, Y chromosome-like; Dicer1, Dicer 1, ribonuclease type III; Cs, Citrate synthase; Tet1, Ten-eleven translocation 1; Rora, RAR-related orphan receptor alpha; Csnk1g2, Casein kinase 1, gamma 2; Ggcx, Gamma-glutamyl carboxylase.

### Effects of aging on spermatogonia

Human spermatogonium includes two types, A and B. Spermatogonia are further distinguished into A_dark_ and A_pale_ based on their morphology. A_dark_ can be further divided into _Adark_ with nuclear rarefaction zone (A_dVac_) and A_dark_ without nuclear vacuole (A_dNoVac_). A_dVac_ does not undergo mitosis and is viewed as a spermatogonial stem cell for spermatogenesis, whereas A_dNoVac_ and A_pale_ spermatogonia differentiate and proliferate into B spermatogonia, which enters the spermatogenesis process (59). Thus, spermatogonia ensure that spermatogenesis happens normally. Studies have indicated that spermatogonia are sensitive to aging. With the increase in age, spermatogonia undergo cell number decrease, cell shedding, nuclear fragmentation, and chromatin agglutination (60–62). A study has reported that with advancing age, the proliferation activity of spermatogonia increases and the spermatogenic efficiency decreases (63), which might contribute to reproduction-related aging phenotypes, such as a decrease in sperm number. Spermatogonia were labeled using MAGEA4 (64), proliferating spermatogonia using PCNA (65), and round spermatids using CREM (66). While the proportion of PCNA-positive spermatogonia increased compared with the young group, the proportion of CREM-positive round spermatids to PNCA-positive spermatogonia per seminiferous tubule decreased, which indicates a decline in the function of spermatogonia, namely spermatogenic efficiency (63). This effect of age on spermatogonia may be attributed to several mechanisms, one of which is the DNA damage theory of aging and the loss of quiescence. With age, the times of DNA replications in the spermatogonia clusters increase and the risks for replication errors increase (for example, the spermatogonia of a 15-year-old boy experiences 35 replication cycles, whereas those of a 40-year-old man undergo 610 cycles) (67). During spermatogenesis, the key factors of the receptor tyrosine kinase pathway in the proliferation/differentiation balance undergo alterations, and spermatogenesis is increasingly susceptible to mutations as per the selfish-spermatogonial selection model. These mutant spermatogonia are prone to DNA damage, which leads to spermatogonia loss, impaired function, increased apoptosis, decreased spermatogenic efficiency, and consequently increased compensatory proliferation (32, 68–71). With respect to the loss of quiescence, which has been extensively studied in aging in other systems, it is tempting to speculate that mechanisms of loss of quiescence may also exist in the testis. A study has demonstrated that fibroblast growth factors (FGFs) play a critical role in the homeostasis of spermatogonial stem cells (SSCs) (72). Using mouse models, it has been shown that SSC homeostasis is achieved by competition for limited FGFs. In this context, with advancing age, FGFs gradually interfere with the homeostatic regulation of SSCs, thus leading to the loss of SSC quiescence (16).In recent years, multiple positive and negative regulators of spermatogonial senescence have been continuously discovered, which have deepened our understanding of the mechanisms of physiological spermatogonial aging and pathological spermatogonial premature aging. These insights have provided new targets for further research on antireproductive aging and treatments for aging-dependent infertility. The abnormal changes of mRNA, ncRNA, alternative splicing and histone modification in SSCs help understand mechanism of reproductive senescence in aging mice, such as the differential expression of lncRNA *Fendrr* and the decrease of histone modification H3K27me3 deposition (73). A mutation in or reduced expression of *Foxp3* has been shown to result in decreased proliferative capacity and increase the levels of apoptosis in human SSCs, which suggests the positive regulatory role of *Foxp3* in the regulation of reproductive aging (74). Additionally, mir-9a expression has been reported to be significantly upregulated in aging Drosophila testes and enhance the proliferative capacity of SSCs by upregulating N-cadherin expression (75). miRNA-122-5p inhibits apoptosis and promotes the proliferation of early SSCs by competitively regulating CBL expression with lncRNA *CASC7*. This finding signifies that miRNA-122-5p is a positive regulator of spermatogonial senescence although lncRNA *CASC7* is a negative regulator. With advances in gene therapy and germline-targeted drug delivery systems, these noncoding RNAs may hold promise as novel tools or targets for the treatment of testicular premature aging by targeting CBL (76).

### Effects of aging on spermatocytes

Spermatocytes undergo two meiotic divisions to form spermatids with half the number of chromosomes. With increasing age, spermatocytes tend to show an increase in apoptosis and a decrease in cell number (77). Ultrastructurally, age-related changes are seen in the organelles of spermatocytes, such as spiral changes in the endoplasmic reticulum (ER). Furthermore, intranuclear inclusions and multinucleated spermatocytes with a fusion of membranes from multiple spermatocytes are formed (71). Compared with 6-month-old mice, 24-month-old mice demonstrate increased spermatocyte apoptosis, accompanied by changes in apoptosis related gene expression profile, including upregulation of *Gpr116* and *Lin52* and downregulation of *Gpr107* and *Mrc1*, where these genes are associated with germ cells apoptosis (78). Moreover, oxidative stress-related proteins are altered in aged human testes (79).

Apart from changes in the number and cytoplasm of spermatocytes, abnormalities also occur in meiosis. With increasing male age, the risk for meiotic errors increases, triggering meiotic arrest and cell death. These events result in low sperm counts (68, 69). Increased paternal age could lead to the failed pairing of homologous chromosomes, abnormal recombination, and chromosome segregation defects (80). SYCP3, a component of the synaptonemal complex that indirectly indicates the level of chromosomal recombination, has been observed to an age-related increase in recombination rate variation (80). Similar results have been found in men over the age of 45 (81). In mice, the expression of BubR, a spindle assembly checkpoint protein during meiosis, decreases progressively in the testes with advancing age, which lowers the meiotic chromosome segregation accuracy in spermatocytes and results in the appearance of aneuploid gametes. BubR1 mutation can also lead to the early accumulation of senescence-associated molecules, such as P53, P21, P16, P19, and β-galactosidase, thus accounting for testicular premature aging (33).

DNA methylation-related molecules are present in spermatocytes and regulate the meiotic processes (82). For example, the maintenance of DNA methylation by DNMT1 and Np95 regulates chromosome synapsis and promotes homologous chromosome pairing. On the contrary, defects in the above-mentioned proteins lead to abnormal synapsis and affect the meiotic process (83). Because DNA methylation is involved in the aging process of reproduction, abnormalities in it may also affect spermatocyte aging. Age-related meiotic abnormalities can pave the way for chromosome nondisjunction, splitting errors, or partial structural exchange, thus resulting in aneuploid gametes (84). The possible cause for these phenomena is the lifelong occurrence of mitosis and meiosis during spermatogenesis, which exposes the germ cells to higher risks for chromosomal damage, recombination errors, and gene conversion (85). If the chromosome is abnormal, the embryo formed after the egg–sperm union has a high probability of chromosomal abnormalities, thus leading to abortion or other adverse effects, such as trisomy 21 syndrome of paternal origin (86).

DNA damage in the spermatocytes is also an important factor in aging (32). Increased age leads to changes in gene expression related to the DNA repair pathway of germ cells, which lowers the DNA damage repair ability. In aging rats, the pathways of base excision repair (BER) and nucleotide excision repair (NER) in pachytene spermatocytes are differentially altered, with the upregulation of the NER genes and the downregulation of the BER genes (31). In addition, the increase in DNA oxidative damage response in the spermatocytes of superoxide dismutase type 1 (SOD1) KO mice has been reported to exhibit an age-related change, thereby causing reproductive premature aging (87). Hence, it is clear that aging affects the fate of the spermatocytes, but the specific impact and mechanism of pathological premature aging on meiosis needs to be further clarified. By doing so, we can intervene or prevent aging-dependent infertility at the cellular and even molecular levels in the future.

### Effects of aging on spermatids

In the late period of spermatogenesis, spermatids constantly deform to spermatozoa, a process that involves the compaction of the nucleus, the appearance of the acrosome, the formation of the flagellum, and the disappearance of the cytoplasm, etc. Spermatid deformation presents age-dependent features, including acrosomal abnormality, excessive nuclear membrane, the appearance of intranuclear inclusions, excessive droplets in the cytoplasm, and irregular shape of the nucleus (71). Morphologically, multinucleated spermatids appear in the testes of older individuals (71), the specific cause of which might be the cell membrane fusion of adjacent spermatids, which results in cell degeneration (88). In addition, aberrant morphology has been noted in the spermatozoa of 24-month-old hamsters. The acrosomal matrix and nuclear membrane were abnormal, mitochondria were absent, and flagella were curved (89). These findings suggest that the spermiogenesis process is affected by physiological aging, thus inducing the occurrence of sperm malformations. Another important change in spermatid deformation is the removal of excess organelles and cytoplasm, a process in which autophagy has been suggested to play an important role. One of the functions of autophagy is to degrade and recycle the excess proteins and organelles in the cells for reuse. Impaired autophagy is one of the hallmarks of aging. The autophagy-related gene *Atg5* regulates male reproduction, and its absence leads to germ cell abnormalities, as shown by the abnormal deformation of the elongating spermatozoa at stages 10–14, fusion of the elongating spermatozoa, sloughing of germ cells, and the appearance of malformations in the sperm head and tail (24). Owing to the relevance of ATG5 in aging, it can be speculated that autophagy may be involved in progressive infertility. Mammalian target of rapamycin (mTOR) signaling is one of the most critical regulatory pathways for autophagy, and evidence supports that promoting autophagy can alleviate aging-related dysfunction (90). For example, feeding folic acid (FA) could promote spermatogenesis in aged roosters. Furthermore, mechanistic studies have revealed that dietary FA supplementation is a potent anti-testicular aging agent that activates autophagy *via* modulation of mTOR signaling to delay testicular senescence (91). Autophagy gene changes have been widely observed in aging-related studies in other systems. However, in the reproductive system, the crosstalk among sperm deformation, aging, and autophagy is unclear and needs further research. The formation of sperm acrosome is an important stage in spermiogenesis, which is precisely and orderly regulated by various genes, including *Pick1* and *Fads2* (92). Studies have shown that *Pick1* (93) and *Fads2* (94) expressions are decreased in the testis of aged mice or rats, which indicates that genes involved in acrosome formation are also affected by aging. However, whether physiological aging gives rise to abnormal acrosome biogenesis in the spermatozoa by affecting the expression of these genes or whether the deficiency in these genes causes spermatid premature aging needs to be clarified.

## Aging and sperm

After further maturation in the epididymis and female reproductive tract, the sperm binds with the egg to complete fertilization. During this process, the sperm is continuously enhanced in terms of motility and orderly complete capacitation, hyperactivation, acrosome reaction, and fertilization. However, adverse alterations gradually appear in semen volume, sperm motility, and sperm function with advancing paternal age. Daily sperm production is negatively correlated with male age and decreases by more than 30% in men older than 50 years (95). One study found that men’s semen volume decreases by 0.22 mL for every 5 years of age (10) and that the sperm count begins to decrease significantly from the age of 41 years (96, 97). Similarly, sperm total motility and forward motility decrease with increasing male age (97, 98). Sperm motility decreases by 1.2% every 5 years (10), and progressive motility is two-fold lower in men aged 50 years or older compared with those aged 40–50 years (96). During sperm capacitation, a progressive decrease in the tyrosine phosphorylation of sperm protein has been observed with increasing age *in vitro* (99). With regard to sperm fertilization ability, the fertilization rates decrease by 0.3% for each additional year of paternal age (100). Among couples with male partners over the age of 45 years, the men cause a five-fold delay in the women’s ability to conceive (101). Generally, the fertilization rate of men over 55 years of age is 50% lower than that of the young age group, and the conception time is significantly delayed (102).

A study evaluating sperm DNA parameters found that sperms display a typical pattern of aging in different age groups, characterized by a steady increase in sperm telomere length (28). Therefore, to some extent, telomere length can serve as a molecular marker of sperm quality and predict the fertility potential of elderly men. In addition, epigenetic regulations, such as DNA methylation and noncoding RNA regulation, are involved in the functional regulation of aged sperms. With the increase in paternal age, global hypermethylation and local hypomethylation regions increase significantly in the sperm, which is contrary to the age-related DNA methylation characteristics of the somatic cells (85). In a study of common carp (*Cyprinus carpio*) spermatozoa, whole-genome bisulfite sequencing was used to compare DNA methylation in young and aged spermatozoa. The researchers found an increase in DNA methylation in the aged spermatozoa, which was inversely correlated with sperm quality and fertilization rate in the aged common carp (34). As DNA methylation is considered to be one of the key mechanisms in aging (103), a study suggested that the DNA methylation signature of the human sperm can be used to predict age (104). Furthermore, miRNAs are involved in the aging process of the sperm. The expression of miR-125a-5p is significantly upregulated in the aging sperm and is negatively correlated with sperm DNA integrity (105). Additionally, miR-574 impairs the mitochondrial function of the aging sperm by inhibiting ATP production (106).

Oxidative stress refers to an imbalanced state of reactive oxygen species (ROS) and antioxidant levels, which tends to enhance oxidation. Oxidative stress plays a pertinent role in the etiology of male infertility in humans and other mammals (107). High levels of ROS in the sperm can lead to male infertility *via* lipid peroxidation, DNA damage, enzyme inactivation, and protein oxidation. With increasing age, there is a concomitant increase in ROS levels in the semen. Aged spermatozoa are more susceptible to oxidative stress, which may be one of the important causes of aging-related impaired fertility. Various indicators are available to evaluate the level of sperm oxidative stress. ROS oxidizes guanine to 8-hydroxy-20-deoxyguanosine (8OHdG) (108), and the 8OHdG level is a reliable indicator of the degree to which the sperm has endured oxidative stress (67). In addition to 8-OHdG and ROS, total antioxidant capacity (TAC), malondialdehyde (MDA), and glutathione (GSH) are commonly used to evaluate oxidative stress in the aging sperm. The static oxidation-reduction potential (sORP) reflects the imbalance between oxidation and antioxidation and is expected to serve as a new indicator of reproductive aging because the level of sORP in the semen is positively correlated with male age (109).

**Figure 2:**
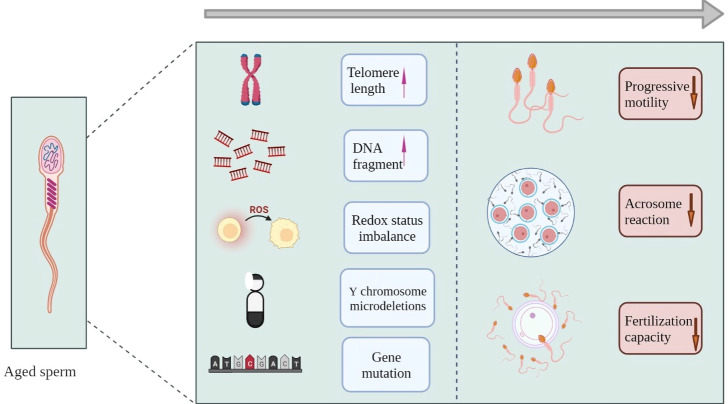
Aging and sperm. In mechanism, aging sperm shows growing telomere length, increased DNA fragmentation, redox imbalance and so on. Eventually in the phenotype, sperm showed abnormal acrosome reaction, decreased fertilization ability and other forms.

Sperm DNA fragmentation index (DFI) is a common parameter used to assess the quality of semen samples (110). Many studies have found that DFI increases with age (111–114). Men over 45 years of age exhibit higher DFI and lower DNA stability (115), and the DFI of 60-year-old men is more than double that of 20-year-old men (116). However, mature sperms possess damage repair mechanisms for defective DNA. In addition, it has been shown that chromosomal defects are closely associated with aging. In mouse models, increasing age has been observed to exacerbate the adverse alterations caused by the deletion of the long arm of the Y chromosome, thus leading to premature germ line senescence (29)(**Figure 2**).

The calcium signal is vital for sperm vitality, capacitation, motility, hyperactivation, acrosome reaction, and fertilization (117). Specifically, the relationship between sperm motility and calcium signaling response levels has been demonstrated (118). The sperm-specific cation channel CatSper is a key channel that mediates calcium ion influx and regulates calcium signaling. Defects in CatSper can cause sperm dysfunction and infertility. A significant decrease in sperm calcium levels in the aging white shrimp *Litopenaeus vannamei* has been reported to result in reduced sperm quality and abnormal acrosome reaction (119). We speculate that the decrease in sperm viability and motility in older males may be related to the imbalance in the regulation of CatSper-mediated calcium signaling. Supplementation with selenium, vitamin-E and Escanbil (*Calligonum*) extract can increase sperm motility and improve reproductive performance in aging mice by upregulating the expression of CatSper channel proteins (120–122).Many gene mutations cause abnormal morphology, reduced sperm number, as well as sperm dysfunction, thus contributing to aging-dependent male infertility. Male conditional KO peroxiredoxin 6(*Prdx6*) mice have been observed to exhibit an age-dependent reduction in sperm count, activity, motility, and fertility. The older mice displayed increased sperm DNA fragmentation, more oxidative damage, enhanced sperm DNA compaction, and decreased sperm proteins, which indicate the protective role of *Prdx6* in male reproductive premature aging (45). The sperm-specific thioredoxin system plays a vital role in the regulation of sperm function, such as thioredoxin domain-containing proteins (TXNDC). *Txndc*-deficient mice show an age-dependent decrease in sperm activity and motility compared with the wild-type mice. In addition, ROS production demonstrates an age-dependent increase in the *Txndc*-KO mice (42). Collectively, these observations allude that testicular premature aging caused by gene mutations, especially alterations in sperm motility and function, may shed new light on the diagnosis and treatment of aging-dependent infertility in clinical practice.

## Fertility, embryo quality and foetal/offspring health

Increased paternal age is associated with poor embryo quality, high risk of miscarriage, stillbirth, low birth weight, short life expectancy and unhealthy childhood outcomes, such as congenital birth defects and achondroplasia, autism, schizophrenia and certain types of cancer (67).

A cohort study has shown that fathers who give birth over the age of 40 have an increased risk of early childhood death (114). Older fathers have poorer quality sperm DNA compared to younger fathers, with an increased proportion of DNA replication errors in embryos and gradual accumulation of chromosomal mutations, leading to poorer embryonic development outcomes (67). Phospholipase C (PLC) is an important sperm factor that induces oocyte calcium oscillations and is necessary for normal embryonic development. A study has found that the increasing age of male mice leads to decreased levels and abnormal localization of sperm PLC (123). Moreover, after the fertilization of the egg by sperm from older men, the developmental capacity of the preimplantation embryo is reduced (124), which may be due to the impaired ability of the senescent sperm to trigger calcium oscillations. Decreased methylation of the transcriptional regulatory regions in the aging sperm genome can be transmitted to the offspring, thus causing the offspring to exhibit abnormal phenotypes similar to those of the parents (125). In aging mouse models, hypomethylation of sperm DNA causes fluctuations in the expression of REST/NRSF (RE1-silencing transcription factor/neuron-restrictive silencer factor) target genes, leading to neurodevelopmental defects in the offspring, exhibiting behavioral phenotypes of neurodevelopmental disorders (126). Therefore, we believe that there is a close relationship between hypomethylation of aging sperm DNA and abnormal behavior of offspring. Metabolism also changes in the offspring of older male mice, manifested as reduced glucose intolerance, liver fat accumulation, increased adipocytes, and impaired energy balance (127). Giving birth at an appropriate paternal age can reduce the risk of these diseases in future generations.

## Aging, sertoli and leydig cells

Sertoli and Leydig cells together play an essential role in maintaining the spermatogenetic microenvironment. Sertoli cells are located on the basement membrane of the seminiferous tubule. These cells form the blood-testis barrier and secrete androgen binding proteins and inhibin, thus providing support and nutrition to the germ cells and promoting sperm maturation and release. Leydig cells, which occur in groups distributed in the seminiferous tubules, secrete androgens, promote spermatogenesis and male reproductive organ development, and maintain secondary sexual characteristics.

Many studies have established that aging damages the morphology and function of Sertoli cells. Their numbers are reduced in 50-year-old men compared with 30-year-old men (128). It has been reported that the Sertoli cells in aged rats lack the cell pseudopodia that engulf the residual body (129). Moreover, the nucleus is extremely irregular, the original localization is lost, the ER is vacuolated, and the lysosomes are exceedingly atypical. These morphological changes damage the spermatogenic microenvironment (130). Functionally, inhibin production by Sertoli cells is reduced (131). Follicle-stimulating hormone (FSH) stimulates the Sertoli cells to produce inhibin, which provides negative feedback to the hypothalamus to inhibit further FSH production and secretion. Hence, disturbance of inhibin damages the hypothalamic–pituitary–gonadal axis, thereby resulting in disorders of the reproductive system hormones. Certainly, the reduction in inhibin is caused not only by chronological aging but also by multifaceted factors; nevertheless, age is a contributor that cannot be ignored and may be mechanistically linked to oxidative damage (132).

A variety of genes regulating Sertoli cell aging have been identified. Normal expression of factors, such as nuclear factor-E2-related factor 2 (*Nrf2*), LIM and cysteine-rich domains 1 (*Lmcd1*), and insulin-like 3 (*insl3*), can relieve damage due to Sertoli cell aging. Icariin alleviates the damage to the Sertoli cells caused by aging mainly by upregulating the ERα/NRF2 signaling pathway (133). LMCD1 regulates the Sertoli cell aging *via* the NFAT1/TXLNA signaling pathway (134). Loss of the Sertoli cell-derived conserved growth factor Insl3 causes testicular premature aging in zebrafish, with a significant increase in germ cell apoptosis after 9 months (135). Moreover, mice with Sertoli cell-specific KO γ-glutamyl carboxylase (GGCX) present late-onset infertility accompanied by the appearance of multinucleated and apoptotic germ cells in the seminiferous tubules. These mice show a significant reduction in the concentration and number of spermatozoa. Mechanistically, GGCX disrupts the junction between the Sertoli cells and the germ cells by affecting the expression of gap junction protein connexin 43 (136). Adverse alterations in these signaling pathways accelerate reproductive senescence, thereby leading to testicular premature aging.

Similar results have been observed in aging Leydig cells, such as decreased number, aberrant cell morphology, intracellular lipid droplets, lipofuscin deposition, and cell senescence (130, 137). Alterations in androgen levels caused by morphological abnormalities in the Leydig cells are noteworthy, and previous studies have shown that plasma testosterone levels decline with age (138). In men, testosterone levels start declining from the age of 30 years, which reduces the activity of Sertoli cells, Leydig cells, and spermatogonia, thereby reducing spermatogenesis (10, 96). Hormonal changes affect gene expression in the aging germ cells. Studies have found that genes related to steroid hormone syntheses, such as *Cyp17a1*, *Cyp11a1*, and *Klk1b27*, exhibit age-related changes (139).

Pathologically, it has been reported that genes are involved in regulating the premature aging of Leydig cells. TAZ plays an important role in the aging of mouse testis, and its deficiency accelerates the apoptosis and senescence of Leydig cells. These changes result in severe structural abnormalities, abnormal expansion of Leydig cells, and decreased fertility. The downregulation of TAZ causes an oxidative stress-induced aging-associated increase in SA-βgal (β-galactosidase) and p21 expression. Compared with the control group, the positive staining of SA-βgal in the Leydig cells has been reported to increase with age (35). The cholesterol-binding protein TSPO (translocator protein), which is localized on the outer mitochondrial membrane, is significantly reduced in aged Brown Norway rats. This protein can significantly enhance testosterone production after its activation by ligands in the Leydig cells (140). Consistent with this observation, TSPO KO mice also exhibit aging-dependent androgen deficiency (141). These findings illustrate that TSPO is essential for the interstitial cells to maintain normal levels of steroidogenesis during testicular premature aging. Results from studies on *Nrf*, a gene involved in the regulation of oxidative stress, have indicated that the levels of testosterone production are significantly reduced in aged KO mice. This finding implies that an imbalance in the antioxidant defense mechanisms of the Leydig cells is also involved in aging-related androgen production abnormalities (142). CDGSH iron-sulfur domain 2 KO mice present the phenotype of reproductive premature aging because the functions of the Leydig cells are hindered. This observation also suggests that a young testicular microenvironment is extremely important for normal reproductive function in aging models (143). These investigations provide insights into the diagnosis of testicular premature aging in humans.

## Rescue and prevention of reproductive aging

Various exogenous and endogenous factors could contribute to testicular aging, such as smoking and gene mutations (67). In addition to studying testicular aging, testicular premature aging, and the underlying mechanisms, there is an increased need to explore how these studies can be used to reduce aging and premature aging of the reproductive system. Such studies can aid in preventing adverse reproductive outcomes, thus contributing to healthy human reproduction. The utilization of middle-aged animals or the construction of reproductive premature aging models to study the anti-reproductive aging effects of medicines, especially antioxidants, is one of the common approaches. A recent study has established that the intranasal administration of nerve growth factors could rescue fertility and restore impaired spermatogenesis in aged male mice (144). *Astragalus membranaceus* and *Punica granatum* are long-lived Chinese herbal medicines in ancient China. Studies on rats of different age groups have shown that *Astragalus membranaceus* telomerase activator-65 and pomegranate supplements could rebuild hormonal balance and testicular structure, exhibit antioxidant activity, and prevent male sterility caused by aging (145). Therefore, they are expected to be applied in clinical therapeutics for reproductive aging and reproductive premature aging. Researchers have employed the testicular premature aging mice model built using D-galactose and sodium nitrite to explore the antiaging effect of BaZiBuShen. The results indicate that BaZiBuShen could improve oxidative stress-induced spermatogenesis disorder in aging mice. Furthermore, it could restore the dynamic balance of redox by upregulating the level of serum TAC and GSH/GSSG (reduced glutathione/oxidized glutathione), downregulating the levels of serum MDA and 8-OHdG (23), and disturbing the SIRT6/p53 and SIRT6/NF- κB signaling pathway. Exogenous supplementation with vitamin D3 or Korean red has been shown to enhance the antioxidant effect, thus resulting in improved spermatogenic capacity in aged rats (146, 147). In addition, exogenous adiponectin treatment has been reported to significantly improve the testicular size, cell proliferation, insulin receptor expression, glucose uptake, antioxidant enzyme activity, and testosterone synthesis in aged mice (148). Melatonin supplementation could also rescue testicular aging caused by the loss of retinoic acid receptor-related orphan receptor alpha (53).

**Figure 3:**
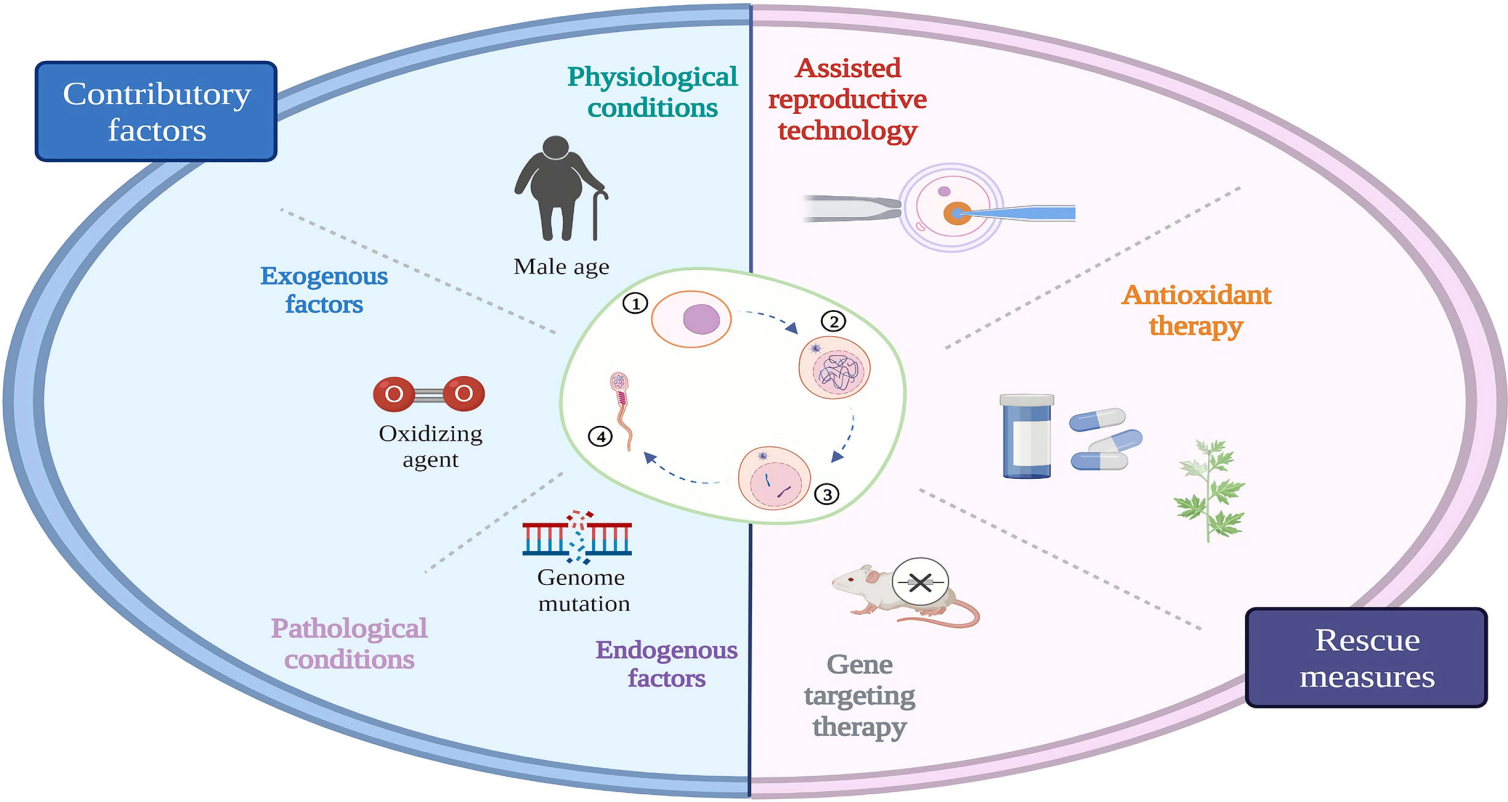
Reproductive aging and rescue. Physiological and pathological factors such as aging, smoking caused testicular aging can be rescued by assisted reproductive technology, antioxidant or gene targeting therapy. ①Spermatogonium; ②spermatocyte;spermatid; ④ sperm.

Targeting the specific genes is another strategy for the treatment of testicular premature aging. Numerous studies have been performed on animal models, which can serve as a reference for future clinical applications. Using a sin-10 pro-oxidant to construct a model of oxidative damage, researchers have found that SOD1 exerts an antioxidant effect in the germ cells of aging mice (46). Overexpression of regucalcin protein also significantly alleviates the oxidative stress level and improves the sperm quality in aging rats (149). Testis-specific paralog of ribosomal large subunit protein RPL39 is required for mouse spermatogenesis. In the absence of RPL39L, mouse spermatogenic cells are less efficient at regenerating themselves and are more likely to degenerate with age (150). Transcription factor TCF21 expression in the mesenchymal cells of the testis is essential for the homeostatic maintenance of testicular somatic cells (151). Hence, RPL39L and TCF21 hold promise as potential targets for the treatment of aging-related infertility. GGCX in the Sertoli cells plays an important role in mouse infertility. A conditional GGCX KO mouse has shown that the expression levels of the proapoptotic gene *Bax* and the antiapoptotic gene *Bcl2* are altered with increasing age and that the mice become infertile in an aging-dependent manner. This infertility could be rescued by the overexpression of connexin 43 (55). Icariin could upregulate ERα/NEF2 signaling, thereby alleviating the damage of the Sertoli cells due to aging (133). Reproductive premature aging caused by incomplete proline catabolism could be ameliorated by limiting the first step in proline catabolism upstream of ALH-6 or by antioxidant treatment (152). Moreover, phosphorylated constitutive Dicer (50), basonuclin 1 (48), and *Nrf2* (153) are potential targets for the treatment of testicular premature aging. For older men, assisted reproductive technologies (ART) are sometimes used clinically to meet their fertility requirements. There are many controversies about the application of ART to aging men, but the study suggest that the sperm of older men do not affect the results of ART (154). In summary, the following three preventive and protecting strategies can be employed for the treatment of aging in different situations (**Figure 3**): 1) Testicular premature aging caused by exogenous stress factors can be treated by avoiding or reducing their exposure and adding prospermatogenic dietary supplements. 2) For testicular premature aging due to genetic mutations, ART can be preferentially selected in cases where gene therapy is not practically feasible. 3) Furthermore, antioxidative drugs can be prescribed for preventing and delaying the aging process based on animal model evidence and clinical experience.

## Conclusions and prospects

The phenotypes of physiological and pathological testicular aging have been well studied; however, the related causes, mechanisms, and rescue measures remain largely unknown. Furthermore, studies on senescence mechanisms in humans are not only limited by ethical requirements but also by the long experimental cycles. Also, because of the discrepancies in the regulation of the reproductive system between different species, the findings on testicular premature aging in animal models are not necessarily appropriate for humans. Therefore, we propose that the next research directions regarding testicular aging and premature aging should be focused on the following aspects: 1) Clinical samples of human testicular aging and premature aging should be directly used to explore the causes and mechanisms *via.* 2) Novel-specific biomarkers should be explored for the early clinical diagnosis of reproductive aging. 3) The feasibility of applying the findings from animal models to human testicular premature aging must be explored. 4) Efficacy should be tracked in intervention outcomes for reproductive aging. 5) Relevant research should be conducted in large-scale clinical cohort to examine the association between reproductive aging and multiple reproductive outcomes. 6) The use of animal models for targeted gene therapy provides references for the prevention and treatment of human testicular premature aging. Systematic and intensive studies are likely to aid in enhancing the reproductive health of men. It is hoped that in the future, it would be possible to treat premature or aging-dependent infertility caused by specific etiologies or genetic mutations *via* precisely targeted therapies.

## Author contributions

SD contributed to literature searching and wrote the manuscript. CC, JZ, and YG designed figures and a table. XHZ and XNZ supervised the study and revised the manuscript. All authors contributed to the article and approved the submitted version.

## Funding

This research was supported by the National Natural Science Foundation of China (81871201), Jiangsu Innovation and Entrepreneurship Talent Plan (JSSCRC2021543), and Large Instruments Open Foundation of Nantong University (KFJN2249).

## Conflict of interest

The authors declare that the research was conducted in the absence of any commercial or financial relationships that could be construed as a potential conflict of interest.

## Publisher’s note

All claims expressed in this article are solely those of the authors and do not necessarily represent those of their affiliated organizations, or those of the publisher, the editors and the reviewers. Any product that may be evaluated in this article, or claim that may be made by its manufacturer, is not guaranteed or endorsed by the publisher.
